# Mutations in *MSH5* in primary ovarian insufficiency

**DOI:** 10.1093/hmg/ddx044

**Published:** 2017-02-08

**Authors:** Ting Guo, Shidou Zhao, Shigang Zhao, Min Chen, Guangyu Li, Xue Jiao, Zhao Wang, Yueran Zhao, Yingying Qin, Fei Gao, Zi-Jiang Chen

**Affiliations:** 1 Renji Hospital, Shanghai Jiao Tong University School of Medicine, Shanghai 200001, P.R. China; 2 Center for Reproductive Medicine, Shandong University, Center for Reproductive Medicine, Shandong Provincial Hospital Affiliated to Shandong University, National Research Center for Assisted Reproductive Technology and Reproductive Genetics, The Key laboratory for Reproductive Endocrinology (Shandong University), Ministry of Education, Jinan, Shandong 250021, P.R. China; 3 State Key Laboratory of Stem Cell and Reproductive Biology, Institute of Zoology, Chinese Academy of Sciences, Beijing 100101, P.R. China

## Abstract

Primary ovarian insufficiency (POI) is a genetically heterogeneous disorder that occurs in familial or sporadic fashion. Through whole exome sequencing in a Chinese pedigree with POI, we identified a novel homozygous missense mutation (ENST00000375755: c.1459G > T, p.D487Y) in the MSH5 gene in two sisters with POI. The homologous mutation in mice resulted in atrophic ovaries without oocytes, and *in vitro* functional study revealed that mutant MSH5 impaired DNA homologous recombination repair. From sanger sequencing of MSH5 in 200 sporadic POI patients, we identified three heterozygous mutations (ENST00000375755: c.1057C > A, p.L353M; c.1459G > T, p.D487Y and c.2107 A > G, p.I703V). Considering the heterozygous p.D487Y carrier in the POI pedigree was fertile, the causality of the three heterozygous mutations in POI need more evidence. Our studies confirmed that perturbation of genes involved in DNA damage repair could lead to non-syndromic POI. The underlying mechanism-inability to repair DNA damage-will receive increasing attention with respect to POI.

## Introduction

Primary ovarian insufficiency (POI), also known as premature ovarian failure (POF [MIM 311360]), is a hypergonadotropic disorder characterized by cessation of menses before 40 years of age and elevated level of follicle stimulating hormone (FSH) (1). Approximately, 1% of women in childbearing age are affected (2). POI is heterogeneous in etiology, including chromosomal abnormalities and single gene mutations, as well as autoimmune, metabolic, infectious and iatrogenic factors. While evidence from genetic factors, provided by population and candidate gene studies, is responsible for the pathogenesis of about 25% of cases, most cases remain unexplained (1). Recently, some novel causative genes have been identified by whole exome sequencing (WES) in POI pedigrees, such as *HFM1* (MIM 615684), *STAG3* (MIM 608489), *MCM8* (MIM 608187), *MCM9* (MIM 610098) and *CSB-PGBD3* (MIM 609413) (3–7). Interestingly, all of these genes are involved in DNA repair or meiosis, which thus proposes a plausible brand-new concept for POI pathogenesis-inability to repair DNA damage.

*MSH5* (MutS homologue 5) is a member of the MutS family, which is principally linked to mismatch repair (MMR). Among all the MSH homologs identified in eukaryotes, the MSH4-MSH5 heterodimers play an important role in homologous recombination (HR) repair for DNA double strand breaks (DSBs) (8). Meiotic crossing-over is processed by SPO11-dependent DSB and HR, hence, MSH5 is also involved in stabilizing and protecting the meiotic recombination intermediate (9). Here, we present an autosomal recessive causative mutation in *MSH5* responsible for two sisters with POI in a Chinese non-syndromic kindred.

## Results

### Homozygous missense mutation in *MSH5* identified in POI pedigree

**Figure 1:**
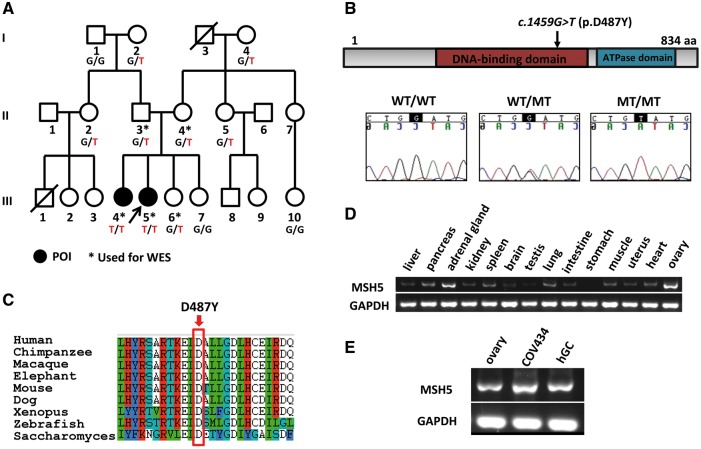
Pedigree of a family with two daughters afflicted by POI and homozygous *MSH5 p.D487Y* variant. (**A**) The pedigree of the index family, ascertained through III5. WES was performed on the family members labeled with asterisk, and those labeled with genotypes were available for Sanger sequencing. “T” denotes the mutant *MSH5* allele, and “G” wild type. Arrow indicates the proband. (**B**) The location of p.D487Y variant is in the DNA-binding domain of MSH5, and the residue is conserved from saccharomyces to human (**C**). (**D**) shows the mRNA level of MSH5 in fetal tissues, which is significantly higher in ovary than others. (**E**) The RT-PCR in fetal ovary, human granulosa cells (hGCs, obtained from one patient receiving *in vitro* fertilization treatment) and COV434 cells, shows that MSH5 is also highly expressed in adult granulosa cells. MT, mutant; and WT, wild type.

Two sisters (III4 and III5) from the non-consanguineous Han Chinese family (Fig. 1A), aged 31 and 29 years, experienced oligomenorrhea since menarche (14 and 13 years old), and amenorrhea occurred approximately 10 years later (Table 1). Both of them have elevated serum FSH, infantile uteri, and atrophic ovaries devoid of follicles. Chromosomal abnormalities, *FMR1* premutation, autoimmune disorders, previous ovarian surgery or chemo-/radiotherapy were absent in any of the family members.

**Table 1:** Clinical features of familial and sporadic POI patients with mutations in *MSH5*

Patient No.	*MSH5* Mutation		Geno- type	Age (year)	Menstrual History		FSH (IU/L)	Uterus (cm ×cm)	Left Ovary (cm ×cm)	Right Ovary (cm ×cm)	Age at Diagnosis (year)	Prior Hormonal Treatment	Childbearing
	sequence variation	Amino acid variation			Age at Menarche (yr)	Age of Amenorrhea (year)							
**Index Family**													
**III4**	c.1459 G>T	p.D487Y	TT	31	14 oligomeno-rrhea	25	44.46	3.5 × 2.7	1.3**×**0.4 Follicle: 0	1.6**×**0.4 Follicle: 0	28	Yes^a^	None
**III5**	c.1459 G>T	p.D487Y	TT	29	13 oligomeno-rrhea	23	94.2	2.5 × 2.1	Invisible	Invisible	25	Yes^a^	None
**Sporadic POI**													
**Case 1**	c.1057 C>A	p.L353M	AC	38	16 oligomeno-rrhea	29	69.64	3.6 × 2.5	Invisible	Invisible	32	Yes ^a^	None
**Case 2**	c.1459 G>T	p.D487Y	GT	32	13 oligomeno-rrhea	19	87.61	3.8 × 2.7	1.4 × 0.9 Follicle: 0.1cm×2	Invisible	28	Yes ^a^	None
**Case 3**	c.2107 A>G	p.I703V	AG	37	14	24	127.4	2.5 × 1.1	Invisible	Invisible	33	Yes ^a^	G1P0L0A1, amenorrhea occurred after induced abortion

^a^1–2 mg daily dose of oral estradiol valerate tablet for 21 days plus oral micronized progesterone (200 mg/days) for 12 days each month.

In this POI pedigree, two affected siblings (III4, III5), both parents (II3, II4), and one unaffected daughter (III6) had been chosen to perform WES (Fig. 1A). In an autosomal-recessive inheritance model, 2 nonsynonymous homozygous variants in *MSH5* (MIM 603382, chromosome 6p21.33) and *ZNF391* (*Zinc Finger Protein 391*, chromosome 6p22.1) were revealed. Sanger sequencing confirmed that neither of the two homozygous variants were present in unaffected family members and both of the variants were absent in 400 fertile women. The *ZNF391* variant (ENST00000244576: c.187A > G, p.S63G) was predicted to be benign by Polyphen2, and the Serine residue mutated was not conserved among species (Supplementary Material, Fig. S1). Furthermore, *ZNF391* has not been related to any human disease and no mutation was found in 200 sporadic patients with POI. Therefore, the *MSH5* variant (ENST00000375755: c.1459G > T, p.D487Y) remained as the only potential candidate for this POI family. Sanger sequencing for *MSH5* in sporadic cases with POI identified 3 additional heterozygous mutations (ENST000 00375755: c.1057C > A, p.L353M; c.1459G > T, p.D487Y and c.2107 A > G, p.I703V), which had not been reported in either the Exome Variant Server or 1000 Genomes database. Among them, p.L353M and p.D487Y located in the DNA-binding domain and the original residues were highly conserved among species from yeast to human, while p.I703V occurred at the less conserved residue located at the ATPase domain (Supplementary Material, Fig. S2).

### Expression of MSH5 in the primate ovary

Through RT-PCR in various tissues of human fetuses, which were induced abortion at 21 weeks, we found MSH5 was highly expressed in fetal ovary and adrenal gland (Fig. 1D). We also found MSH5 was highly expressed in adult human granulosa cells (hGCs), including the hGCs obtained from one patient receiving *in vitro* fertilization treatment and COV434 (human ovarian granulosa tumor cell line) cells (Fig. 1E).

### Homozygous mutant mouse model carrying *Msh5 p.D486Y* point mutation displayed POI phenotype

**Figure 2:**
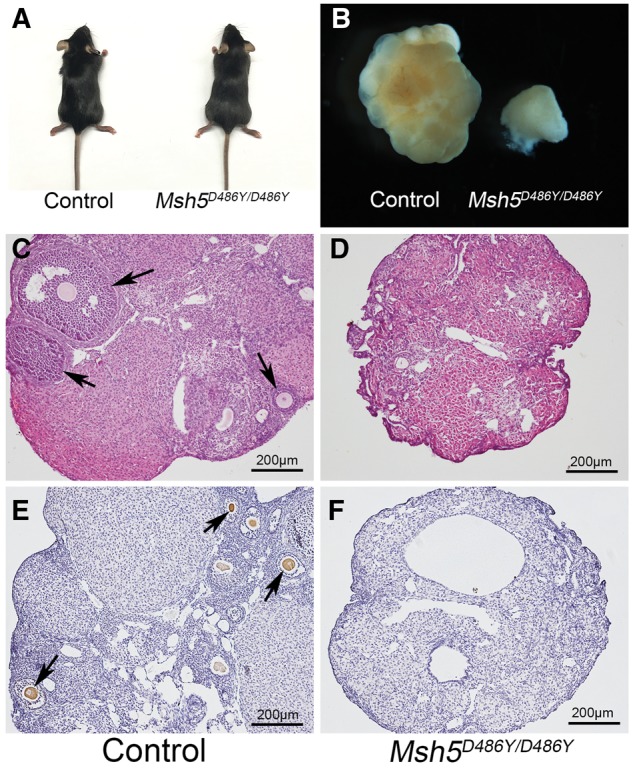
Germ cell loss in ovaries from *Msh5^D486Y/D486Y^* mice. (**A**) The homozygous mutant mice were viable at birth and no obvious developmental defects were observed in adults. (**B**) The size of ovaries from homozygous mutant females was dramatically reduced compared to the control ovaries. The follicles at different developmental stages (arrows) were observed in control ovaries (**C**), whereas no developing follicle was noted in *Msh5^D486Y/D486Y^* ovaries (**D**). Numerous Ddx4-positive germ cells (arrows) were observed in control ovaries (**E**), but no germ cells were noted in *Msh5^D486Y/D486Y^* ovaries (**F**). Scale bars: 200 μm.

The homologous residue for human *MSH5* c.G1459 (ENST00 000375755: p.D487) in mouse is *Msh5* c.G1456 (EN SM UST00000007250: p.D486), which is highly conserved (Fig. 1C). To examine the functional effect of p.D487Y identified in human POI, the mouse model carrying *Msh5 p.D486Y* point mutation was generated using a CRISPR/Cas9 system (Supplementary Material, Fig. S3A and B). The homozygous mutant mice *Msh5^D486Y/D486Y^* were obtained by intercrossing of *Msh5^+/D486Y^* mice (Supplementary Material, Fig. S3C). The homozygous mutant mice were viable at birth and no obvious developmental defects were observed in adults (Fig. 2A). However, all the *Msh5^D486Y/D486Y^*females were infertile. The size of the ovary was dramatically reduced compared to that of control females (Fig. 2B). The results of histological studies showed that the follicles at different developmental stages (black arrows) were observed in control ovaries (Fig. 2C), whereas no developing follicle was noted in *Msh5^D486Y/D486Y^* ovaries (Fig. 2D) at 2 month of age. Numerous Ddx4-positive germ cells (black arrows) were observed in control ovaries (Fig. 2E), but no germ cells were noted in *Msh5^D486Y/D486Y^* ovaries (Fig. 2F).

### Adverse effect of MSH5 p.D487Y on DNA repair for DSBs

**Figure 3:**
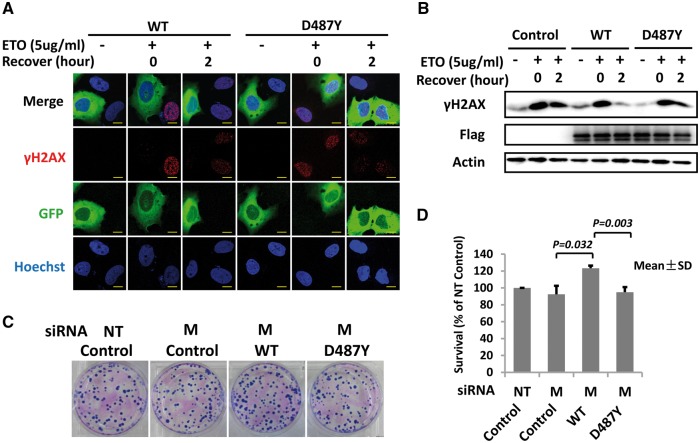
MSH5 p.D487Y impaired DNA repair. (**A**) Immunofluorescence showed the γH2AX foci formation in U2OS cells overexpressing wild type (WT) or mutant (D487Y) MSH5-GFP protein when suffering from ETO treatment. Scale bars: 5μm. (**B**) The γH2AX concentration among U2OS cells overexpressing blank vector (Control), wild type (WT) or mutant (D487Y) MSH5-Flag protein was detected by western blot. The expression of wild type and mutant MSH5 was detected by Flag antibody and β-actin was used as the loading control. (**C**) and (**D**) showed the clonogenic survival rate of WT- and D487Y-overexpressing cells in response to ETO treatment. The experiments on U2OS and HeLa cells both had 3 replicates independently. Data in the figure are shown as mean ± SD. M, siRNA targeting at MSH5; NT, non-targeting siRNA; and WT, wild type.

DSBs were induced by ETO treatment, and γH2AX foci were observed at the DSBs sites, which gradually disappeared along with the repair processing (Fig. 3A). The result showed that γH2AX level was much higher in the U2OS cells overexpressing mutant MSH5 than wild type (Fig. 3B). Furthermore, HeLa cells overexpressing mutant MSH5 showed a lower clonogenic survival rate than wild type, indicating the adverse effect of p.D487Y on DNA repair capacity or cellular resistance for DSBs. (Fig. 3C and D).

## Discussion

In a Han Chinese family having two siblings with non-syndromic POI, we identified an autosomal recessive mutation in *MSH5* (p.D487Y) by WES, which impaired DNA repair for DSBs. Furthermore, the mice carrying homologous mutation present with POI phenotype, indicating that p.D487Y in *MSH5* might be the causative mutation of POI pathogenesis.

Oocyte genome is particularly vulnerable to ubiquitous external damage, such as DSBs and single-strand breaks (SSBs). Ability to repair DNA damage is essential to maintain the supply of oocytes necessary for reproduction. DSBs are likely the most detrimental damage, normally repaired by HR and non-homologous end joining (NHEJ) (10,11). The MSH4-MSH5 heterodimer is one of the crucial factors in HR by recognizing Holliday junctions and forming a hydrolysis-independent sliding clamp that embracing adjacent homologous duplex arms (8).

In the *Msh5^-/-^* female mice, meiosis is arrested due to the disruption of chromosome pairing at prophase I, subsequently, resulting in progressive loss of oocytes and infertility (12). Mandon-Pépin *et al.* identified a novel heterozygous mutation p.P29S (2/41, 4.9%) in the *MSH5* gene in 41 women with POI (13); however, functional validation studies were lacking. In the current study, the homozygous mutation p.D487Y identified is located at a position that is highly conserved; surprisingly the variation of its homologous residue in yeast (Msh5 p.D527A) has been reported to result in poor spore viability (14,15). In our study, the *Msh5^D486Y/D486Y^* mice were infertile with atrophic ovaries, confirmed the adverse effect of human MSH5 p.D487Y on oogenesis. Moreover, human cells overexpressing MSH5 p.D487Y showed diminished survival rate and remaining more DSBs unrepaired compared with cells overexpressing wild type MSH5, indicating the impaired function in the process of HR repair. As shown by RT-PCR, besides fetal ovary, MSH5 was also expressed in granulosa cells. These results suggested that perturbations in MSH5 might not only disturb meiosis in oocytes, but also affect HR repair in granulosa cells during mitosis, followed by accelerated follicle loss through atresia, finally causing POI.

To determine the contribution of *MSH5* gene to sporadic POI, we screened *MSH5* by Sanger sequencing in 200 sporadic cases with POI and identified 3 heterozygous mutations (ENS T00000375755: c.1057C > A, p.L353M; c.1459G > T, p.D487Y and c.2107 A > G, p.I703V). The carriers of p.L353M and p.D487Y suffered from oligomenorrhea since menarche occurred, whereas the carrier of p.I703V experienced amenorrhea at age of 24 just after spontaneous pregnancy. While mutation p.I703V located at the ATPase domain, mutations p.L353M and p.D487Y located at the DNA binding domain of MSH5 and were highly conserved among species (Supplementary Material, Fig. S2). However, they might not be responsible for POI pathogenesis due to heterozygosity. In support of this assumption, the mother of the proband (II4) in the POI pedigree, carried heterozygous p.D487Y as well. She gave birth to four children and had no sign of POI or early menopause. Our data suggest that the biallelic causal variants in *MSH5* are uncommon in idiopathic POI.

Together with the findings in *HFM1, STAG3, MCM8, MCM9* and *CSB-PGBD3*, our studies in *MSH5* further confirmed that perturbation of genes involved in DNA damage repair could lead to non-syndromic POI. The underlying mechanism-inability to repair DNA damage-is plausible and will receive increasing attention with respect to POI.

## Materials and Methods

### Exome sequencing and data analysis

Genomic DNA was extracted from peripheral blood leukocytes using DNeasy Blood & Tissue Kit (Qiagen). Exome sequences were captured with SureSelect Target Enrichment System for Illumina Paired-End Sequencing Library (Agilent Technologies), and DNA sequencing was performed on the Illumina platform (Illumina HiSeq). Reads were mapped to the hg19 reference genome, and variants were called and annotated using Genome Analysis Toolkit (GATK), ANNOVAR, and custom pipelines. Candidate variants were confirmed in other family members and 400 control women. Sanger sequencing of candidate genes in 200 independent sporadic patients with POI were performed to detect other variations in those genes.

### Detection of MSH5 expression in primate ovary

The mRNA level of MSH5 was tested by RT-PCR in ovary, uterus, heart, brain, lung, stomach, liver, kidney, spleen, testis, pancreas, adrenal gland, intestine and muscle tissues of human fetuses (induced abortion at 21 weeks), as well as in hGCs from one patient receiving *in vitro* fertilization treatment and COV434 cells.

### Knock-in mouse model

The mouse model carrying *Msh5 p.D486Y* point mutation was generated using CRISPR/Cas9 system, the homozygous mutant mice *Msh5^D486Y/D486Y^* were obtained by intercrossing of *Msh5^+/D486Y^* mice.

### DNA damage and recovery assay

The human osteosarcoma U2OS cells were transiently transfected with wild type or mutant MSH5-GFP or MSH5-Flag plasmids, and were incubated in media containing Etoposide (ETO) (5μg/ml) for 1 h at 37°C to induce DSBs, followed with normal media replacement and recovery for 2 h at 37°C. Phosphorylation of the Ser-139 residue of histone variant H2AX, forming γH2AX, is an early cellular response to DSBs, which here was used as a sensitive marker for DSBs. Immunofluorescence and western blot were performed to detect DNA damage and recovery degree by staining γH2AX. The clonogenic survival rate after ETO treatment was calculated in HeLa cells, in which endogenous MSH5 was silenced with siRNA and then wild type or mutant MSH5 was overexpressed.

### Statistics

Comparisons of clonogenic survival rate between cell lines were calculated using independent sample *t*-test. All the *P* values were two-sided, and *P* <0.05 was considered statistically significant. SPSS 19.0 computer software (IBM, Armonk, NY, USA) was used for data analysis.

### Study approval

The recruitment of POI pedigree, sporadic POI patients and control women from Shandong Provincial Hospital Affiliated to Shandong University was approved by the Institutional Review Board of Center for Reproductive Medicine Shandong University. Written informed consent was obtained from all participating subjects. All animal studies were carried out in accordance with the protocols approved by the Institutional Animal Care and Use Committee at the Institute of Zoology, Chinese Academy of Sciences (CAS).

## Supplementary Material

Supplementary Material is available at *HMG* online.

## Supplementary Material

Supplementary Data
