# Supplementary material for: Mutations in MSH5 in primary ovarian insufficiency
Source: Hum Mol Genet. 2017 Feb 8;26(8):1452–7. doi: 10.1093/hmg/ddx044 (PMC5393145; doi:10.1093/hmg/ddx044)
Supplement: Supplementary Data [file ddx044_Supp.docx]

**
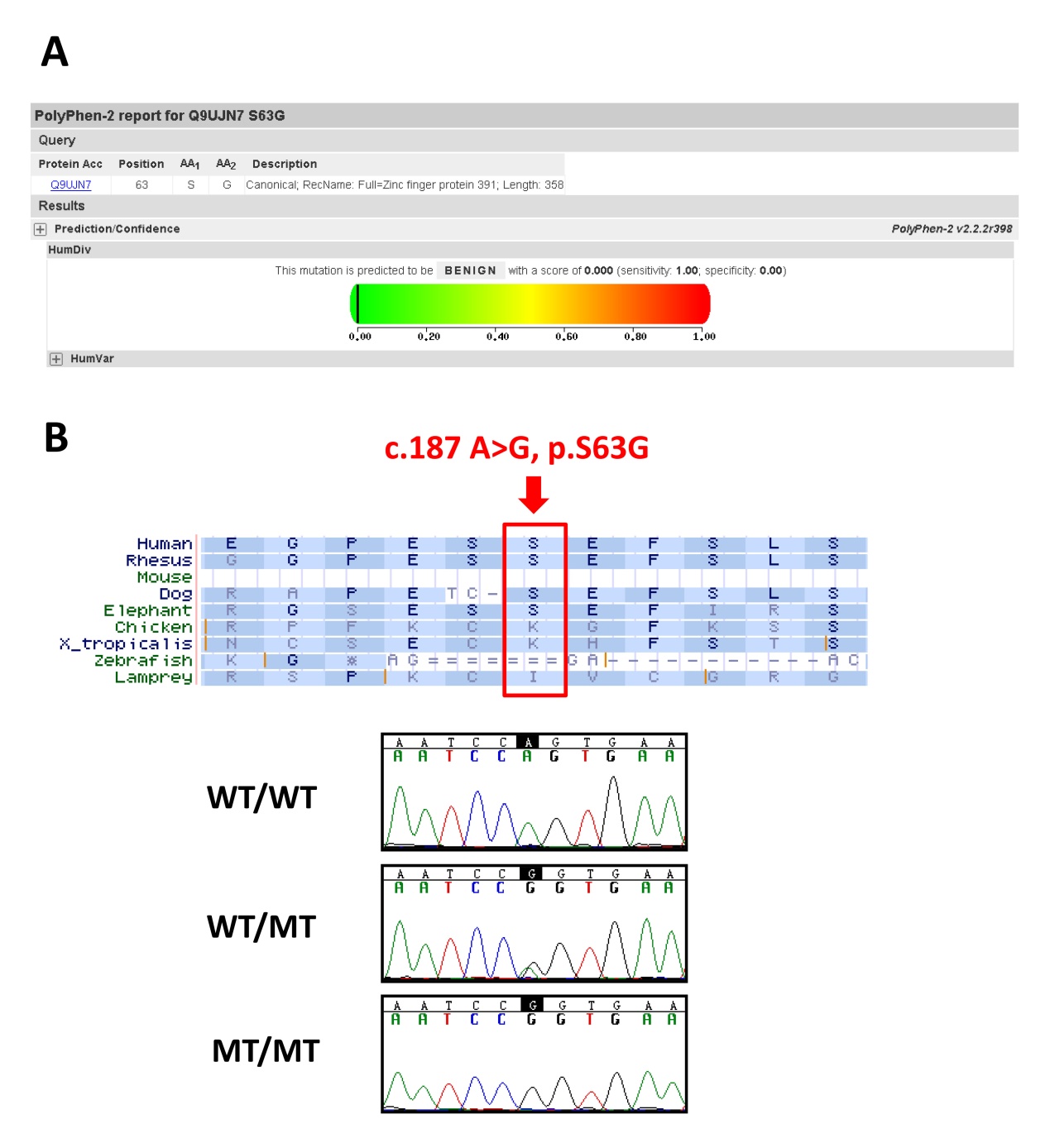
** **Figure S1. Features of *ZNF391* mutation identified in the POI pedigree.**

(A) The *ZNF391* mutation (ENST00000244576: c.187A>G, p.S63G) is predicted to be benign on PloyPhen-2 website. (B) The Serine residue is not conserved among species.

**
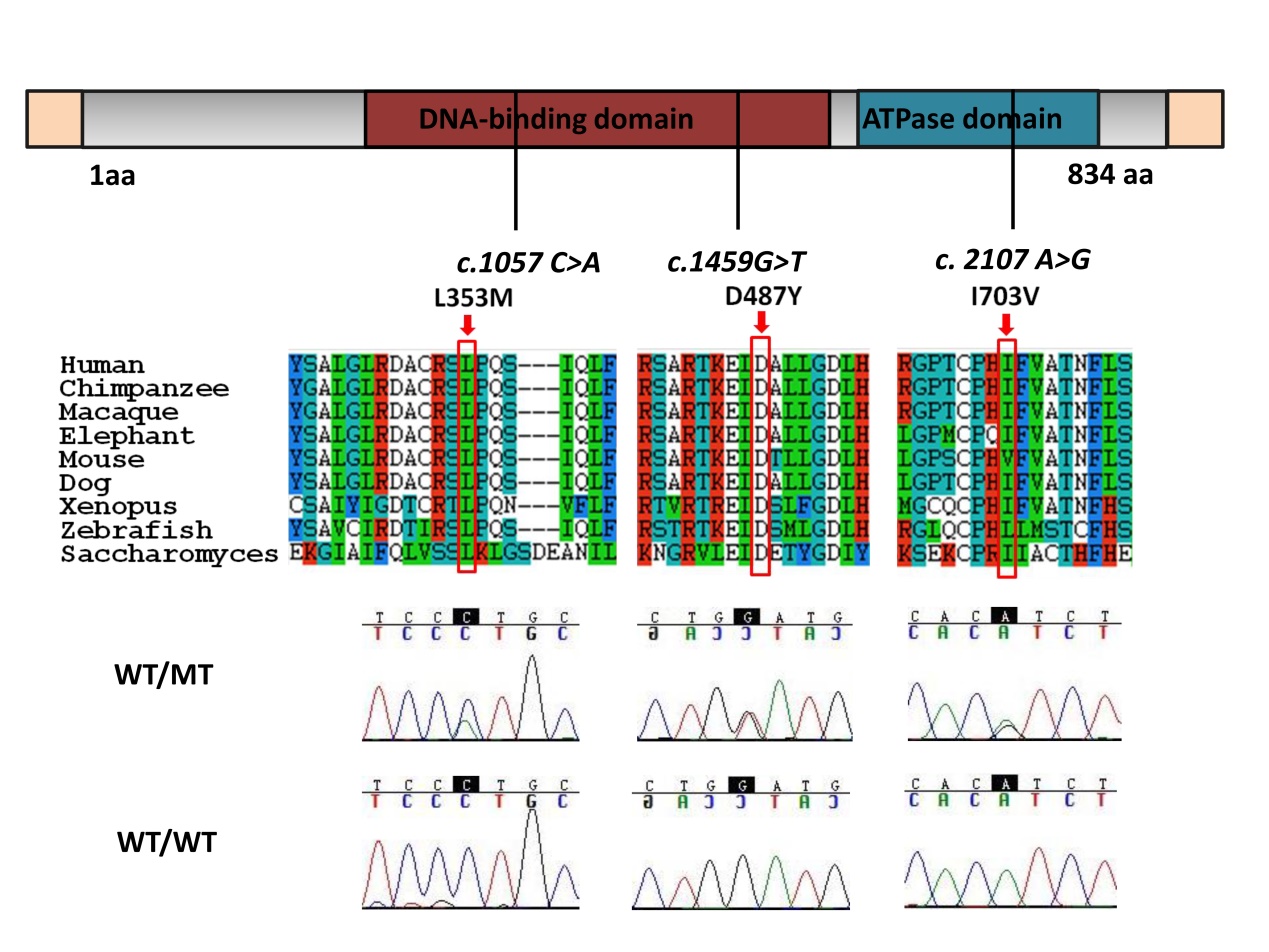
****Figure S2. Heterozygous mutations in *MSH5* identified in sporadic POI patients.**

Mutations p.L353M and p.D487Y located at the DNA binding domain of MSH5, and were highly conserved among species. Mutation p.I703V located at the ATPase domain, and was not conserved as the other two sites.

**
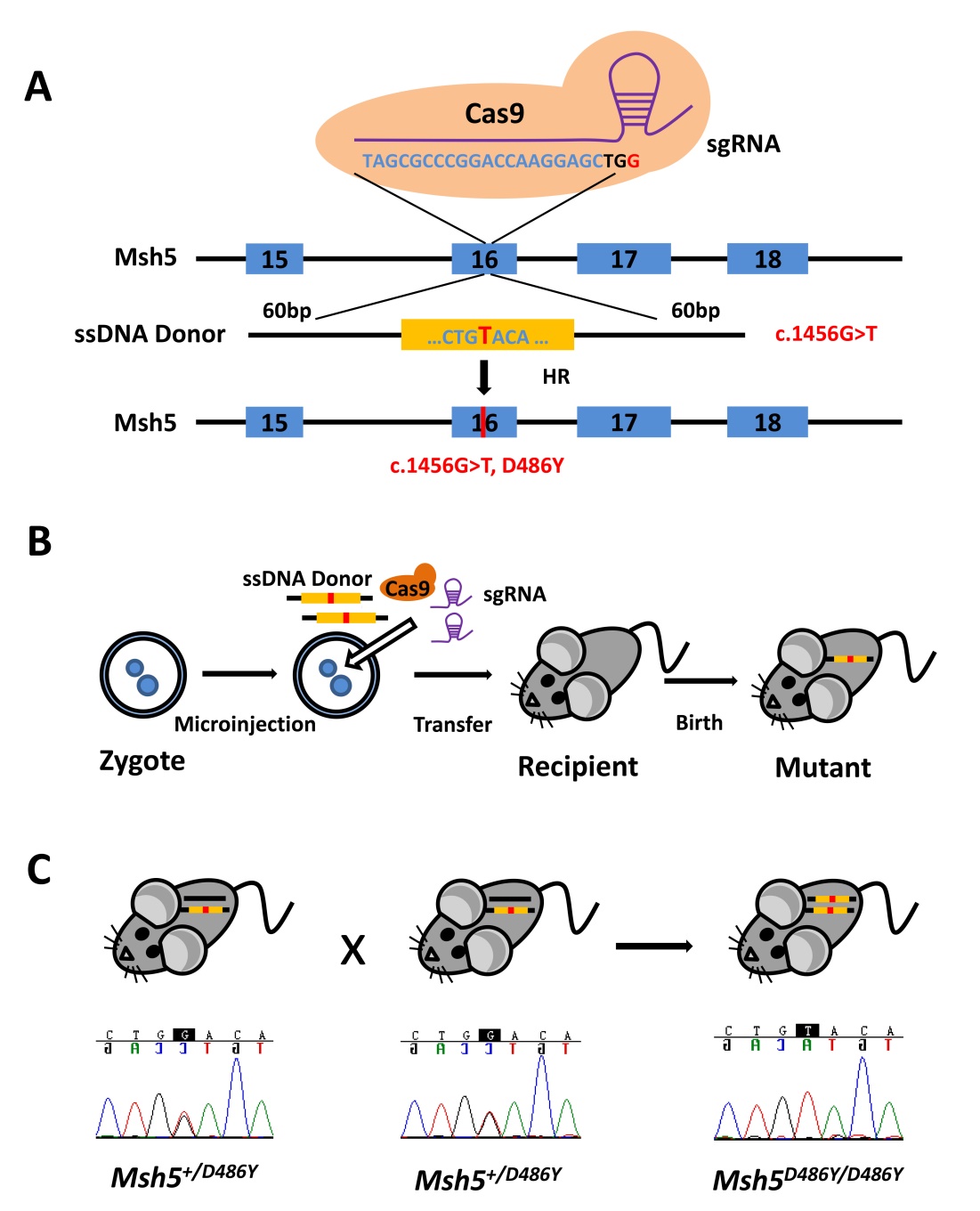
**

**Figure S3. Generation of *Msh5^D486Y/D486Y^* mice with CRISPR/Cas9 system.**

(A) Schematic overview of strategy to generate *Msh5 D486Y* point mutant mouse model. The coding sequence of single-guide RNAs (sgRNA) is capitalized and labeled in blue (“G” is exactly the mutation point, which could prevent the second cut). The ssDNA donor is indicated as 60 bps upstream and 60 bps downstream around the mutated residue, which is labeled in red “T”. The mutation is indicated as a red bar in exon 16. (B) The donor DNA carrying D486Y mutant was microinjected into pronucleus of zygotes, along with Cas9 mRNA and sgRNAs targeting Msh5. Then, the zygotes were transferred into the oviducts of ICR pseudopregnant recipient mice. (C) The knock-in mouse with homozygous D486Y was obtained by crossing of heterozygous D486Y carriers. Genotyping was performed by Sanger sequencing, and the chromatogram was indicated below.

**METHODS**

**Inclusion criteria for sporadic POI patients**

Inclusion criteria for POI consisted of cessation of menstruation occurred before 40 years old, with at least twice serum follicle stimulating hormone (FSH) concentrations exceeding 40 IU/L. Women with chromosomal abnormalities, suffered pelvic surgery, chemotherapy or radiotherapy were excluded. The patients having family member diagnosed with POI were excluded. Information was sought on associated somatic anomalies, specifically adult onset neurologic disorder, referred to as fragile X–associated tremor/ataxia syndrome, or mental retardation. Written informed consent was obtained from all participants. This study was approved by the Institutional Review Board of Reproductive Medicine of Shandong University.

**Detection of MSH5 expression in primate ovary**

**RT-PCR**

The human fetal ovary tissues, as well as uterus, heart, brain, lung, stomach, liver, kidney, spleen, testis, pancreas, adrenal gland, intestine and muscle tissues were obtained from fetuses undergoing pregnancy termination at 21 weeks because of social factors. Human granulosa cells (hGCs) was obtained from one patient receiving in vitro fertilization treatment due to tubal factor. COV434 was the human ovarian granulosa tumor cell line. Total RNA was extracted from the tissues respectively, and reverse transcribed into cDNA with the use of PrimeScript RT reagent Kit with gDNA Eraser (TaKaRa, DRR047A). Then, PCR was performed with the primers for MSH5 coding sequence. The PCR products were detected by 1% agarose gel electrophoresis.

RT-PCR primers for MSH5:

| Primer | Sequence |
| --- | --- |
| F-forward | 5’-TCCAGCTCTTTCGGGACATT-3’ |
| R-forward | 5’-AGGACTTGTGGGGAGTAACG-3’ |

**Immunofluorescence**

U2OS cells were transiently transfected with wild type or mutant MSH5-GFP plasmids using X-tremeGENE HP DNA Transfection Reagent (Roche, 06366236001), and cultured for 24h. Then, the cells overexpressing wild type or mutant MSH5-GFP fusion protein were treated with Etoposide (ETO) (5μg/ml) for 1h at 37^o^C. The media with ETO was removed, and the cells were fixed with 4% PFA immediately or after recovery in normal culture media for 2h at 37^o^C. After being fixed, cells were washed with 1×PBS, and incubated with 0.25% Triton X 100 for 5 min at room temperature. Then, the cells were blocked with 5% BSA for 30 min at room temperature, followed with the incubation with primary antibody against Phospho-Histone H2AX (Ser139) (Cell Signaling Technology, #9718) for 12h at 4^o^C. Then, the cells were incubated with the second antibody labeled with TRITC (Thermo Fisher, A16040) for 1h at 37^o^C. Cell nuclei were stained with Hoechst 33258 (Beyotime, C0003) for 1-5 min at room temperature. Wash the cells with PBS and observe them under the fluorescence microscope.

**Western blot**

U2OS cells were transiently transfected with wild type or mutant MSH5-FLAG plasmids using X-tremeGENE HP DNA Transfection Reagent (Roche, 06366236001), and cultured for 24h. The cells overexpressing wild type or mutant MSH5-FLAG were treated with ETO (5μg/ml) for 1h at 37^o^C. Then, the cells were harvested immediately or after recovery with normal culture media for 2h at 37^o^C. The protein was obtained with the use of Radio Immunoprecipitation Assay (RIPA) buffer (Beyotime, P0013C) with 1mM Phenylmethanesulfonyl fluoride (PMSF) (Beyotime, ST506). Western blot was performed with the primary antibodies against FLAG (Cell Signaling Technology, #2368), Phospho-Histone H2A.X (Cell Signaling Technology, #9718) and ACTIN (Sigma) following the manufacturer's introduction.

**Clonogenic survival**

HeLa cells were cultured and MSH5 was silenced with MSH5 siRNA mix or non-targeting siRNA (Negative control) for 24h. Then, the wild type and mutant MSH5-FLAG-pcDNA3.1 and pcDNA3.1 (as control) plasmids were transiently transfected into the HeLa cells using X-tremeGENE HP DNA Transfection Reagent (Roche, 06366236001)，and cultured for 24h. Then, the cells were exposed to the culture media containing ETO 5μg/ml for 1h at 37℃. After treatment, the cells were washed and harvested, and reseeded into a new 6-well plate by 500 cells per well (three wells for duplicate) and incubated for 10-14 days. Then the colonies was stained with crystal violet and counted. This experiment was replicated three times.

siRNA sequence:

| siRNA name | Primer | Sequence |
| --- | --- | --- |
| Negative control | sense | 5'- UUCUCCGAACGUGUCACGUTT-3' |
|  | antisense | 5'- ACGUGACACGUUCGGAGAATT-3' |
| MSH5-homo-495 | sense | 5'-CCUCUUCCUCUCUUCCAUUTT-3' |
|  | antisense | 5'-AAUGGAAGAGAGGAAGAGGTT-3' |
| MSH5-homo-531 | sense | 5'-CCUCACAGUUCGAGCACUUTT-3' |
|  | antisense | 5'-AAGUGCUCGAACUGUGAGGTT-3' |
| MSH5-homo-624 | sense | 5'-CCUGGGCUUUAAGAAAUUUTT-3' |
|  | antisense | 5'-AAAUUUCUUAAAGCCCAGGTT-3' |

**Generation of Msh5 D486Y point mutant mouse model**

The mouse model carrying Msh5 D486Y point mutation was generated using CRISPR/Cas9 system.

The coding sequence of single-guide RNAs (sgRNA) targeting at Msh5 is:

“TAGCGCCCGGACCAAGGAGCTGG” (The last base “G” is exactly the mutation point, which could prevent the second cut). The DNA template for gRNA was obtained by PCR with primers：

| Primer | Sequence |
| --- | --- |
| F-forward | 5’-GGATCCTAATACGACTCACTATAGGTAGCGCCCGGACCAAGGAGCGTTTTAGAGCTAGAAATAGC-3’ |
| R-forward | 5’-AAAAGCACCGACTCGGTGCC-3’ |

The PCR product was purified and transcribed into mRNA using MEGAshortscript™ T7 Transcription Kit (Catalog #:AM1354), then the sgRNA was purified with life Technologies MEGAclear™ Transcription Clean-Up Kit (Catalog #:AM1908) and ready for microinjection.

The ssDNA donor is designed to contain 60 bp homology arms on each side flanking the mutation (ENSMUST00000007250: c.1456G>T, p.D486Y, labeled in red and underlined), which was synthesized and ULTRAPAGE purified. The sequence of ssDNA donor is:

TGCACCCAGTTTCTCTCAGAGGACAAGCTGCACTATCGTAGCGCCCGGACCAAGGAGCTG**T**ACACGCTGCTGGGAGACCTGCACTGCGAGATCCGGGGTGAGGAGCCCGTGGTAGGAGGGG

Then, Cas9 mRNA, sgRNAs and ssDNA donor with mutation D486Y were microinjected into the pronuclear of zygote from C57 mice. Following injection, the zygotes were transferred into the oviducts of ICR pseudopregnant recipient mice. The offsprings were genotyped by Sanger sequencing with primer:

| Primer | Sequence |
| --- | --- |
| F-forward | 5’-AGGTGGGTCTCTGTGAGTTG-3’ |
| R-forward | 5’-CACCTGCCATTCCTGATTCG-3’ |
